# Herbal Therapies in Functional Gastrointestinal Disorders: A Narrative Review and Clinical Implication

**DOI:** 10.3389/fpsyt.2020.00601

**Published:** 2020-07-10

**Authors:** Yong Sung Kim, Jung-Wook Kim, Na-Yeon Ha, Jinsung Kim, Han Seung Ryu

**Affiliations:** ^1^ Wonkwang Digestive Disease Research Institute, Gut and Food Healthcare, Wonkwang University School of Medicine, Iksan, South Korea; ^2^ Good Breath Clinic, Gunpo, South Korea; ^3^ Department of Gastroenterology, Kyung Hee University College of Medicine, Seoul, South Korea; ^4^ Department of Clinical Korean Medicine, College of Korean Medicine, Graduate School, Kyung Hee University, Seoul, South Korea; ^5^ Department of Gastroenterology, College of Korean Medicine, Kyung Hee University, Seoul, South Korea; ^6^ Brain-Gut Stress Clinic, Division of Gastroenterology, Wonkwang University Hospital, Iksan, South Korea

**Keywords:** herbal, functional dyspepsia, irritable bowel syndrome, STW 5, peppermint

## Abstract

The pathophysiology of functional gastrointestinal disorders (FGIDs) is still unclear and various complex mechanisms have been suggested to be involved. In many cases, improvement of symptoms and quality of life (QoL) in patients with FGIDs is difficult to achieve with the single-targeted treatments alone and clinical application of these treatments can be challenging owing to the side effects. Herbal preparations as complementary and alternative medicine can control multiple treatment targets of FGIDs simultaneously and relatively safely. To date, many herbal ingredients and combination preparations have been proposed across different countries and together with a variety of traditional medicine. Among the herbal therapies that are comparatively considered to have an evidence base are iberogast (STW-5) and peppermint oil, which have been mainly studied and used in Europe, and rikkunshito and motilitone (DA-9701), which are extracted from natural substances in traditional medicine, are the focus of this review. These herbal medications have multi-target pharmacology similar to the etiology of FGIDs, such as altered intestinal sensory and motor function, inflammation, neurohormonal abnormality, and have displayed comparable efficacy and safety in controlled trials. To achieve the treatment goal of refractory FGIDs, extensive and high quality studies on the pharmacological mechanisms and clinical effects of these herbal medications as well as efforts to develop new promising herbal compounds are required.

## Introduction

Functional gastrointestinal disorders (FGIDs) are a group of diseases with variable combinations of chronic or recurrent gastrointestinal (GI) symptoms not explained by structural or biochemical abnormalities (1). FGIDs include diseases that are commonly found in daily practice, such as gastroesophageal reflux disease (GERD), functional dyspepsia (FD), irritable bowel syndrome (IBS), and functional constipation (FC), and show various symptom presentation throughout the GI tract. They impair the quality of life (QoL) of patients and entail a huge expenditure of medical resources (2, 3). Although the possible pathological mechanisms involved in these diseases have been studied and proposed from various perspectives, their etiologies are still not fully understood. Numerous factors such as GI dysmotility, visceral hypersensitivity, altered immune function, stress, central nervous system dysregulation, intestinal dysbiosis, and genetic predispositions seem to affect the clinical expressions of these diseases, but the mechanisms or complex crosstalk between the pathways have not been clearly elucidated (4).

In spite of treatments have been attempted for FGIDs in the past decades by controlling specific etiologies determined from preclinical and human studies, the positive therapeutic yields of these treatments were unsatisfactory in many cases. Various single-drug treatments with a single target site have been used, but cases that showed complete improvement of symptoms are rare, except for a few in which the treatment showed a marginal effect. Despite the advances on the understanding of the pathophysiology of FGIDs and the development of drugs targeting novel pathways, agents that display satisfactory therapeutic effects on various FGIDs according to both patients and physicians are still limited (5).

Herbal medications have been used in many countries by various races since ancient times and are still used by some physicians or as home remedies. They can be used as complementary and alternative medicine for patients with FGIDs when primary therapeutic approaches fail and are recommended by treatment guidelines for FGIDs in certain countries (6–8). As the various FGIDs overlap in many cases or show a wide range of symptoms even if they occur as a single disease entity (9, 10), herbal medications with multiple mechanisms of action in virtue of diverse components can potentially regulate various, complex etiologies simultaneously and comprehensively improve symptoms of FGIDs. Furthermore, herbal medication will have fewer adverse effects owing to its proven safety from long-term use. Thus, it can be a more desirable therapeutic agent for FGIDs.

A variety of herbs can be used as therapeutic agents for FGIDs and are presumed to target the GI system, as proposed by traditional medicines in numerous countries. However, this paper only discusses drugs whose mechanism of action was demonstrated by various preclinical studies and proved effective by clinical trials. Herbs with potential indications not covered in this review require further research and validation.

## Methods

A PubMed literature search was performed using the following terms individually or in combination: STW, STW-5, Iberogast, peppermint, peppermint oil, menthol, Mentha, Rikkunshito, Yukgunja-tang, Liu-Jun-Zi-Tang, DA-9701, Motilitone, esophagus, stomach, small intestine, colon, dyspepsia, nausea, abdominal pain, gastroesophageal reflux disease, esophagitis, irritable bowel syndrome, constipation, and functional gastrointestinal disorders. In the case of motilitone, published in Korean were additionally searched. More than 250 references were initially reviewed. Following removal of references that overlapped between searches and those lacking original data, the authors agreed on inclusion of 111 references based on which the information has been presented within this manuscript. The Jadad score were calculated by two investigators (YSK and HSR) independently to assess the quality of each included study (10).

## Results

### STW-5 (IBEROGAST^®^)

STW-5 is liquid preparation made from extracts of nine well-known herbs, obtained using alcohol and combined at a fixed ratio. It has been used clinically in German-speaking countries for over several decades and is sold in Europe as an over-the-counter medication (11). The liquid extract contains unique constituents, including fresh plant extract of bitter candytuft (*Iberis amara*) and extracts from eight dried herbs, including angelica roots (*Angelicae radix*), chamomile flowers (*Matricariae flos*), caraway fruit (*Carvi fructus*), St. Mary’s thistle fruit (*Cardui mariae fructus*), balm leaves (*Melissae folium*), peppermint leaves (*Menthae x piperitae*), greater celandine (*Chelidonii herba*), and licorice root (*Liquiritiae radix*), generated using a defined extraction method with fixed amounts of components (12). Various plant components display single effects, and their interactions have also been predicted. Therefore, STW-5 has shown therapeutic effects through multiple mechanisms in several preclinical studies and clinical trials for a wide range of symptoms.

#### Mechanisms of Action

##### Effects on Gastrointestinal Motility and Sensation

In an animal study that used stomach muscle strips of guinea pigs, STW-5 was shown to act directly on muscles rather than exert neural mechanisms of action, as it was not affected by concomitant treatment with tetrodotoxin, capsaicin, or N-nitro-l-arginine methyl ester (L-NAME). STW-5 inhibited and relaxed muscle activity dose dependently in the fundus area of the stomach but enhanced the amplitude of phasic contraction in the antrum area (13, 14). In a study with healthy volunteers, administration of STW-5 1.1 ml (20 drops) in nine subjects led to a significant increase in proximal gastric volume measured with a gastric barostat. It also increased the antral pressure wave from antroduodenal manometry using 16-channel catheter in 12 subjects, but did not cause any change in pyloric or duodenal pressures and gastric emptying (GE) measured by scintigraphy (12). In an experiment that used an ileal muscle strip isolated from guinea pigs, STW-5 showed spasmolytic properties by reducing acetylcholine- and histamine-induced contractions. However it increased the basal resting tone and contraction of the atonic segment through its component *Iberis amara* extract. Thus, STW-5 exerted dual activity with spasmolytic and tonic effects depending on the basal tone of the intestine and these effects were also observed in the duodenum, jejunum, and colon (15). In the large and small intestines of mice, STW-5 reduced the amplitude and frequency of slow waves (16). The target sites in the intestine were 5-HT_4_, 5-HT_3_, muscarinic M_3_, and opioid receptors (17).

In an *in vivo* experiment that used Wister rats, extracellular multi-unit intestinal afferent nerve recordings were performed in anesthetized animals. When the afferent nerve discharge was measured after applying stimuli such as ramp distension, bradykinin, or serotonin, STW-5 reduced the afferent nerve discharge that occurred in response to the chemical and mechanical stimuli without affecting the baseline discharge (18).

##### Effects on Inflammation and Mucosal Function

STW-5 reduced the incidence of gastric ulcer and inflammation by reducing the indomethacin-induced acid hyper-production and increasing leukotrienes, mucin secretion, and prostaglandin E_2_ release in Wistar rats (19). Its inhibitory effects on intestinal inflammation were also validated in the dextran sulfate sodium- and trinitrobenzenesulfonic acid (TNBS)-induced colitis models (20, 21). In the Ussing chamber study that used human intestinal tissues, STW-5 exerted a pro-secretory effect by increasing epithelial chloride fluxes through the cystic fibrosis transmembrane conductance regulator and calcium-activated chloride channels, showing therapeutic potential for constipation symptoms (22). 

**Figure 1:**
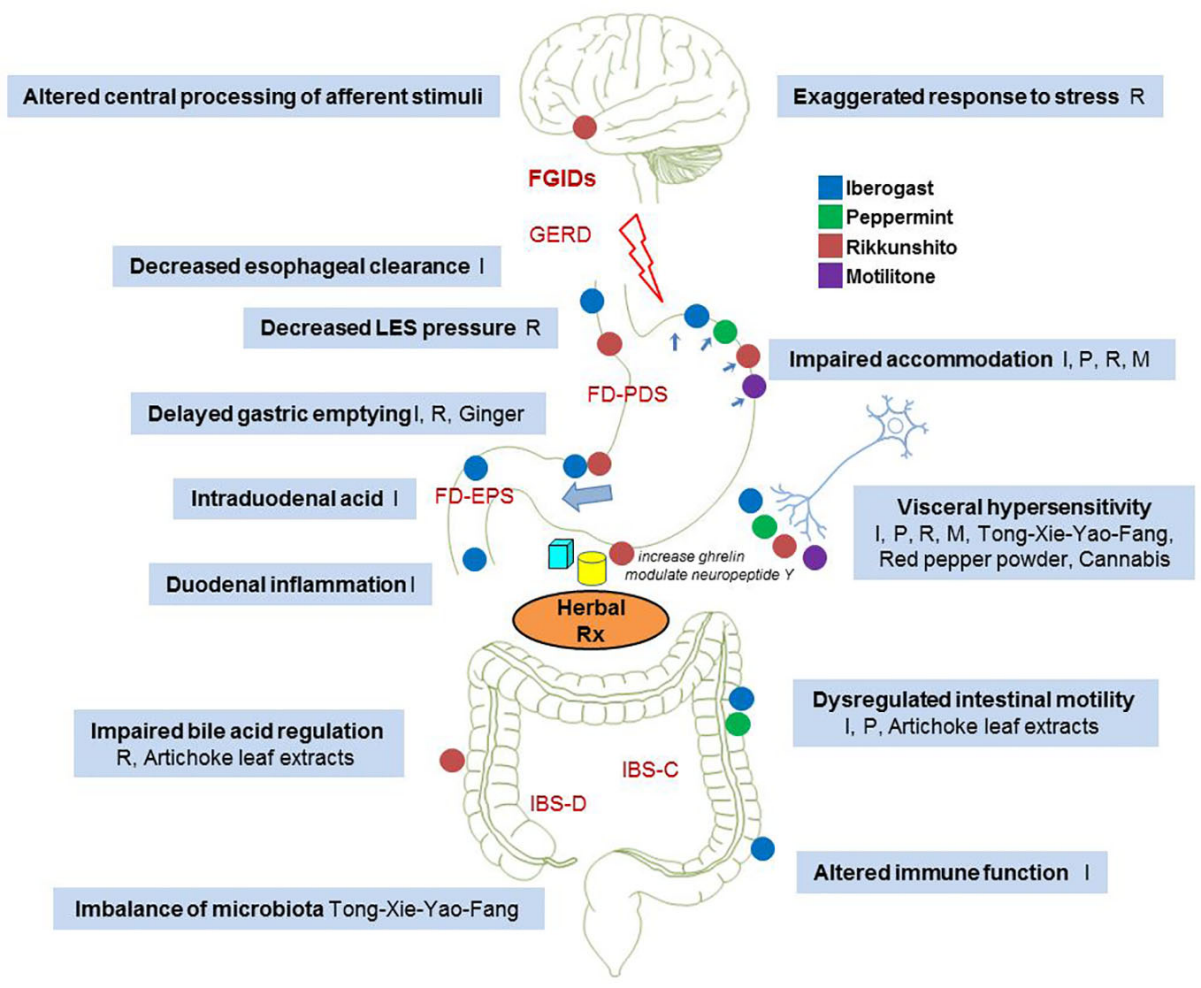
Proposed effects of herbal preparations on various functional gastrointestinal disorders. I, Iberogast; P, Peppermint; R, Rikkunshito; M, Motilitone.

#### Clinical Studies

##### Functional Dyspepsia

In a multicenter placebo-controlled double-blind study conducted in Germany, either STW-5 or placebo was administered in 315 patients with FD by Rome II criteria for 8 weeks after a 7-day washout period. The validated GI symptom (GIS) scale that incorporated 10 dyspeptic symptoms was used to assess symptom severity using a 5-point Likert scale. After 4 and 8 weeks, the GIS score significantly decreased (*p <* 0.05) in the STW-5 group as compared with the placebo, and this effect was independent of *Helicobacter pylori (H. pylori)* status. However, no primary efficacy parameters were used except for the GIS score, and the percentage of responders (based on the improvement of the GIS scores by ≥40%) was very high in the placebo group (78%) (23) (**Table 1**). In a multicenter placebo-controlled double-blind study with 103 patients with FD by Rome II criteria, the GIS score decreased after 4 weeks of STW-5 administration and the percentage of treatment responders by STW-5 was 75%, which was higher than the 54% in the placebo group (*p =* 0.03). However, the difference in mean GIS score was only 2.2 point, and no correlation was observed between symptom improvement and GE measured using the ^13^C-octanoic acid breath test (24).

**Table 1:** Summary of clinical trials of STW-5.

Subject	Design	County	Comparison	Number	Outcome	Ref.	Jadad score
FD by Rome II	Multi-center, randomized, double-blind, placebo-controlled	Germany	STW-5 1.1 ml (20 drops) t.i.d., 8 weeks *vs*. placebo	158:157	Improvement of GIS score (*p* < 0.05) *H. pylori* did not influence the results	von Arnim et al. (23)	5
FD by Rome II	Multi-center, randomized, double-blind, placebo-controlled	Germany	STW-5 1.1 ml (20 drops) t.i.d., 4 weeks *vs*. placebo	44:42	Improvement of GIS score (*p =* 0.08) and the proportion of patients with a treatment response (*p =* 0.03) Effects of STW-5 were not mediated by accelerating GE	Braden et al. (24)	4
IBS by Rome II	Multi-center, randomized, double-blind, placebo-controlled	Germany	STW-5 1.1 ml (20 drops) t.i.d., 4 weeks *vs*. STW-5-II 1.1 ml (20 drops) t.i.d., 4 weeks *vs*. bitter candytuft mono-extract 1.1 ml (20 drops) t.i.d., 4 weeks *vs*. placebo	51:52:53:52	STW 5 and STW 5-II were significantly better than placebo in reducing the total abdominal pain score (STW 5, *p =* 0.0009; STW 5-II, *p =* 0.0005) and the irritable bowel syndrome symptom score (STW 5, *p =* 0.001; STW 5-II, *p =* 0.0003)	Madisch et al. (25)	5

FD, functional dyspepsia; GIS, gastrointestinal symptom; t.i.d., three times a day; GE, gastric emptying.

##### Irritable Bowel Syndrome

In a randomized, double-blind placebo-controlled multicenter trial conducted in Germany, STW-5 and STW-5-II were administered to 208 patients with IBS by Rome II criteria. Changes in total abdominal pain scores and IBS symptom score, which incorporated eight symptoms (flatulence/meteorism, sensation of tension or fullness, sensation of incomplete evacuation, changes in bowel habit) defined by the authors, were measured using a 4-point Likert scale. The results showed that the total abdominal pain score (STW-5, *p =* 0.0009; STW-5-II, *p =* 0.0005) and the IBS symptom score (STW-5, *p =* 0.001; STW-5-II, *p =* 0.0003) improved after 4 weeks (25).

#### Safety

In a clinical study with 315 patients with FD that was conducted in Germany, no significant difference in overall tolerability was observed after the administration of STW-5 for 8 weeks as compared with the placebo group. Adverse effects such as abdominal pain, pruritus, sore throat, alopecia, hypersensitivity, hypertension, and GI pain were observed in five patients (23). In a study with 103 patients with FD, 8 adverse events showed possible or probable relationships, including 3 adverse events (stomatitis, abdominal pain, and diarrhea) in the STW-5 group and 5 adverse events (rhinitis, diarrhea, dyspepsia, vomiting, and genitourinary tract infection) in the placebo group (24). In a study with 208 patients with IBS, only 2 minor adverse events (headache and constipation) were reported, and the treatment was continued (25). However, there were recent reports of severe hepatotoxicity associated with the use of this drug (26, 27), which is possibly related with greater celandine, one of the extracts used in STW-5 (28).

#### Usage

A commercially available STW-5 preparation (Iberogast^®^, Steigerwald GmbH, Darmstadt, Germany) is a dark brown, clear to lightly cloudy liquid supplied in a brown glass bottle and administered at the recommended dose of 1.1 ml (20 drops), three times daily (24, 25).

### Peppermint Oil

Mint plants have a long history of medicinal use as stomach soothers and anecdotal evidence of its purported efficacy abounds to this day (29, 30). Peppermint is a perennial herb (*Mentha* x *piperita*) that grows throughout Europe and North America. Usually, peppermint is a sterile hybrid of two mints, spearmint (*Mentha spicata*) and water mint (*Mentha aquatica*) (31). Peppermint oil (PMO) is obtained by steam distillation from the fresh leaves of peppermint (30). It contains a large number (> 80) of components including menthol (35–55%), menthone (20–31%), menthyl acetate (3–10%), cineol, and several other volatile oils (31–33). Its major constituent and active ingredients appear to be menthol that in nature exists as a pure stereoisomer (32). Owing to the various constituents of peppermint, it has a variety uses, including topical application as an antiseptic and analgesic, inhalation as aromatherapy, and oral formulation for treatment of headache and various FGIDs such as IBS.

#### Mechanisms of Action

##### Effects on Gastrointestinal Motility

Many lines of evidence to date suggest that PMO acts as a smooth muscle relaxant of the GI tract (32). *In vitro* research has indicated that both PMO and its constituent menthol exert calcium channel blocking properties in guinea pig ileal smooth muscle, contributing to intestinal smooth muscle relaxation (34). Another study also suggested that PMO markedly attenuated contractile responses in the guinea pig tenia coli to acetylcholine, histamine, 5-hydroxytryptamine, and substance P (35). They suggested that the PMO relaxes GI smooth muscle by its ability to decrease the influx of extracellular calcium ions through voltage-dependent channels (32, 35). In addition, PMO may affect the enteric nerve system directly. In an experiment using the mouse small intestine, menthol induced membrane potential depolarization in a concentration-dependent manner using cultured interstitial cells of Cajal (ICC), the pacemaker cells of the GI tract (36). The authors also identified that PMO acts on ICC by a G-protein-, Ca^2+^-, Rho-kinase-, COX-, and thromboxane A_2_ dependent manner *via* transient receptor potential ankyrin 1 (TRPA1), which may explain the promoting effect on GI motility.

In addition, there is also evidence indicating that PMO decreases small bowel contractility and attenuates orocecal transit. Both duodenally instilled and given orally PMO decreased duodenal contractions in a double contrast barium study and manometry (37–39). Furthermore, a study using hydrogen breath testing showed that the PMO combination with caraway oil had delayed orocecal transit in healthy volunteers (40). Similarly, PMO decreased colonic spasm and/or peristalsis. A randomized trial of endoscopy evaluated inhibition effect of intraluminally administrated PMO on colonic motility during colonoscopy using the barostat balloon and endoscopic evaluation (41). Although the duration of relaxation was short, about 20 minutes, the authors found peristalsis and spasm of the colon were diminished after administration of PMO.

##### Effects on Visceral Sensitivity

Peppermint (*via* menthol) is a well-known topical analgesic. Some studies show that PMO can attenuate visceral pain in animal models (42, 43). An animal study showed that the combined treatment of peppermint and caraway oil had a significant effect on the reduction of post-inflammatory visceral hyperalgesia in rats that had been pretreated with TNBS/ethanol (43). Recent studies have suggested that the reduction of visceral pain by menthol is mediated through the TRPM8 and/or TRPA1 receptor (42, 44).

##### Effects on Esophageal and Gastric Function

An early study demonstrated that PMO decreased esophageal body and lower esophageal sphincter (LES) pressure based on esophageal manometry in healthy adults (45). Likewise, orally administered PMO reduced spasm of the esophagus in double-contrast barium meal examination (37). Another study using esophageal manometry demonstrated that PMO did not affect the esophageal body and LES pressures in patients with diffuse esophageal spasm despite improvement of manometric findings (46). Given orally or topically sprayed PMO also decreased spasm of the sto mach (37, 47). Some studies using manometry and/or barostat have demonstrated various effects on the gastric physiology such as decreased intragastric pressure, decreased gastric motility index, with no change in gastric accommodation (38, 39, 48). However, studies addressing the effects of PMO on GE have shown mixed results (40, 49).

##### Psychological Effects

Although there have been no studies in humans demonstrating the mood modulating effects, some studies in rodents have suggested that menthol has dose-dependent anxiolytic effects by acting on dopamine signaling pathways (50–52). A study in rodents demonstrated that menthone, a constituent of PMO, promotes ambulation in mice and dopamine might be involved effects inducing mental excitation and thereby reducing mental fatigue (50). Thus, PMO could help change mood by reducing mental fatigue. This may be another mechanism through which PMO activity targets the brain-gut axis in the common pathogenesis of various FGIDs.

#### Clinical Studies

##### Irritable Bowel Syndrome

A prospective, double blind, placebo-controlled randomized trial with 57 patients with IBS by Rome II criteria showed that 64% of patients receiving enteric-coated PMO and 34% of placebo users experienced a reduction in the total IBS symptom score at 4 weeks of ≥50% (*p* < 0.002) in the intention-to-treat population (54) (**Table 2**). They also assessed the symptom score at 4 weeks after the end of treatment (8 weeks) and found a persisting beneficial effect at 8 weeks (*p <* 0.05). Another randomized double-blind placebo-controlled study on 90 outpatients with IBS by Rome II criteria was conducted to demonstrate the efficacy of PMO on QoL (55). Patients were randomly assigned to receive one capsule of Colpermin^®^ (Tillotts Pharma, Ziefen, Switzerland) or a placebo three times daily for 8 weeks. They examined the effectiveness of PMO in terms of relieving symptoms and improving QoL using a questionnaire addressing six IBS symptoms and the 36-item Short Form Health Survey (SF-36) for QoL. At week 8, 42.5% of patients receiving PMO and 22.2% of patients receiving placebo were free from abdominal pain or discomfort (*p <* 0.001). However, there were no significant between-group differences detected in other IBS symptoms such as abdominal distension, flatulence, loose stool, hard stool, urgency, and incomplete evacuation. Although overall scores of SF-36 for the two groups were not significantly different, patients in the PMO group showed improvements in the SF-36 domains of bodily pain, general health, social functioning, and role limitations due to emotional problems.

**Table 2:** Summary of clinical trials of peppermint oil.

Subject	Design	County	Comparison	Number	Outcome	Ref.	Jadad score
IBS based on symptoms	Single center, randomized, double-blind, placebo-controlled	Taiwan	Peppermint oil 137 mg t.i.d. or q.i.d., a.c., 4 weeks *vs*. placebo	25:49	Symptom improvements after peppermint oil therapy were significantly better than after placebo.	Liu et al. (53)	4
IBS by Rome II	Single center, randomized, double-blind, placebo-controlled	Italy	Peppermint oil 450 mg b.i.d., a.c., 4 weeks *vs*. placebo	28:29	A 4-week treatment with peppermint oil is more effective than placebo in reducing abdominal symptoms related to IBS.	Cappello et al. (54)	4
IBS by Rome II	Single center, randomized, double-blind, placebo-controlled	Iran	Peppermint oil 187 mg t.i.d., a.c., 8 weeks *vs*. placebo	33:27	Severity of abdominal pain and discomfort were reduced significantly in the peppermint oil group. Peppermint oil significantly improved the QoL.	Merat et al. (55)	5
IBS by Rome III	Multicenter, randomized, double-blind, placebo-controlled	USA	Peppermint oil 180 mg t.i.d., 4 weeks *vs*. placebo	35:37	Patients treated with peppermint oil experienced greater improvement in multiple individual gastrointestinal symptoms as well as in severe or unbearable symptoms, compared to placebo.	Cash et al. (56)	5
Childhood IBS by Manning or Rome II	Multicenter, randomized, double-blind, placebo-controlled	USA	Peppermint oil 374 mg or 187 mg t.i.d., 2 weeks *vs*. placebo	21:21	After 2 weeks, improvements in the change of symptom scale were reported in 71% of the patients receiving peppermint oil compared with 43% receiving placebo with statistical significance.	Kline et al. (57)	3
FD based on symptoms	Single center, randomized, double-blind, placebo-controlled	Germany	Peppermint oil 90 mg and caraway oil 50 mg b.i.d., 4 weeks *vs*. placebo	48:48	For the major symptoms (intensity of pain, sensation of pressure, heaviness and fullness, and global improvement), the superiority of combination therapy of peppermint oil and caraway oil over placebo was statistically significant.	May et al. (58)	5
FD by Rome III	Multicenter, randomized, double-blind, placebo-controlled	Germany	Peppermint oil 90 mg and caraway oil 50 mg b.i.d., 4 weeks *vs*. placebo	58:56	Compared to placebo, 4-week treatment with peppermint oil and caraway oil therapy significantly reduced symptoms of epigastric pain and postprandial distress and improved the participants’ QoL.	Rich et al. (59)	5

IBS, irritable bowel syndrome; t.i.d., three times a day; q.i.d., four times a day; b.i.d., two times a day; a.c., before meal; QoL, quality of life; FD, functional dyspepsia.

A recent study reported the findings of a 4-week randomized controlled trial which tested a novel formulation of PMO designed for sustained release in the small intestine (IBgard^®^, IM HealthScience, Boca Raton, FL, USA) for its efficacy and tolerability in reducing IBS symptoms in 72 patients with mixed IBS (IBS-M) or IBS with diarrhea (IBS-D) by Rome III criteria (56). The specialized enteric-coating utilized in their trial consisted of a solid-state matrix that was triple-coated and designed to deliver PMO with sustained release to the small intestine with fewer potential adverse effects. At trial completion, there was a 40% reduction in the total IBS symptom score in the PMO group compared to baseline *vs*. 24.3% with placebo (*p =* 0.0246). Moreover, there was an increased improvement in multiple individual GI symptoms, as well as in severe or unbearable symptoms compared to the placebo.

Similarly, some meta-analyses have shown PMO to be effective in IBS, the number needed to treat (NNT) ranged between 2 and 3 (60–62). In most recent meta-analysis of 12 randomized controlled trials with 835 patients with IBS, the risk ratio (RR) for the effect of PMO (n = 253) *versus* placebo (n = 254) on global symptoms was 2.39 (95% CI 1.93–2.97, *p <* 0.00001) (63). Overall, there were no differences in the reported adverse effects, PMO (9.3%) *versus* placebo (6.1%). The NNT with PMO was three for global symptoms and four for abdominal pain.

##### Functional Dyspepsia

To our knowledge, no study has determined whether PMO alone is useful in patients with FD. However, some randomized controlled trials have shown PMO to be effective for FD when used in combination with other herbal remedies such as STW5-II and caraway oil (58, 59, 64). Recently, a randomized placebo-controlled trial with 114 outpatients with chronic or recurrent FD demonstrated that a fixed peppermint and caraway- oil combination (Menthacarin) treatment is effective for the relief of FD symptoms and improvement of disease-specific QoL (59). After 4 weeks of treatment, pain and discomfort scores improved by 7.6 ± 4.8 and 3.6 ± 2.5 points for Menthacarin and by 3.4 ± 4.3 and 1.3 ± 2.1 points for placebo (all *p <* 0.001), respectively.

#### Safety

PMO has been used safely for various conditions in short-term clinical trials. Menthol, the major constituent of PMO, is also listed as generally regarded as safe by the US Food and Drug Administration. In a randomized controlled trial with 90 patients with IBS, the most common adverse events of PMO treatment group were heartburn, headache, and dizziness. However, these were not significantly different in the two groups (55). However, PMO is relatively contraindicated in patients with hiatal hernia or significant GERD, because its effects on the lower esophageal sphincter can lead to exacerbation of symptoms (31). Especially, when non-coated PMO is taken orally, it can cause heartburn, nausea, and vomiting. The effective delivery method to the target organ by enteric coating is believed to prevent or reduce these upper GI symptoms as well as improve PMO efficacy (31). Even in the enteric coated formulation, heartburn could develop because of the premature rupture of capsules containing PMO (41). High concentrations of PMO have been reported to cause anal burning (65). Other minor adverse effects of PMO reported in clinical trials include allergic reactions and blurred vision. Because PMO may inhibit the cytochrome P450 system, it theoretically could lead to increased serum levels of drugs such as amitriptyline, haloperidol, and cyclosporine which are metabolized by this enzyme (66, 67). However, this interaction has not been proven in humans.

#### Usage

The dosage of PMO for the treatment of GI diseases usually ranges from 0.2 to 0.4 ml, three times a day. The oral dosage range studied in most IBS trials was 187 to 500 mg (0.2–0.4 ml) administered two or three times daily for 2 to 8 weeks (53–55). To secure the availability of unmetabolized PMO at the target organ, a lower digestive tract in IBS, enteric-coated formation such as Colpermin^®^ and Mintoil^®^ (Cadigroup, Rome, Italy) capsules have been developed and have been widely used in clinical trials or real-world practice (54, 55). Each capsule of Colpermin® and Mintoil^®^ contains 187 mg and 225 mg PMO, respectively. These formulated capsules are usually administered 30 to 60 min before meals in order to guarantee low gastric pH which prevents untimely capsule dissolution with premature release of PMO into the stomach (54). In FD, most trials used a dose of 90 mg of PMO in combination with 50 mg of caraway oil (58, 59).

### Rikkunshito

Rikkunshito (RKT; *Rikkunshi-to* in Japan, *Yukgunja-tang* in Korea, *Liu-Jun-Zi-Tang* in China) is one of the more famous herbal formulas in traditional medicine. It has been prescribed for hundreds of years to alleviate abdominal discomfort due to indigestion (68). In Japan, RKT has been widely used and marketed to treat various symptoms of the GI tract (69). The Japanese Ministry of Health and Welfare gave RKT approval for medical use (TJ-43, Tsumura & Co., Tokyo, Japan) (70).

RKT is a form of extracted granules for oral intake, containing 4.0 g of dried mixture consisting of eight crude herbs in fixed proportions (71): *Pinelliae* tuber (18.6%), *Ginseng* radix (18.6%), *Atractylodis lanceae* rhizoma (18.6%), *Hoelen* (18.6%), *Aurantii nobilis* pericarpium (9.3%), *Zizyphi* fructus (9.3%), *Glycyrrhizae* radix (4.7%), and *Zingiberis* rhizoma (2.3%) (72, 73). RKT has been shown to reduce GI symptoms including dyspeptic or reflux symptoms, as well as improve fundic relaxation, GE, and antral contractions (74). It has been suggested that RKT shows synergetic effects through a complex interactive pathway by each component (75, 76).

#### Mechanisms of Action

##### Effects on Esophageal Sensory and Motor Function

RKT has been shown to suppress dilation of the intercellular space and to improve the barrier function of esophageal mucosa in an experimental rat esophagitis model (77). In a study with eight children with symptomatic gastroesophageal reflux, a 7-day administration of RKT reduced clinical symptoms and acid exposure time in the distal esophagus in 24-h esophageal pH monitoring tests, by activating esophageal acid clearance mechanisms (78). In a pilot study of 30 patients with proton pump inhibitor (PPI)-refractory non-erosive reflux disease (NERD), 8 weeks of RKT treatment improved esophageal clearance by reducing the residual LES pressure during swallows, and increasing complete bolus transit rate and peristaltic contractions rate in esophageal multichannel impedance and manometry (79).

##### Effects on Plasma Ghrelin Level

RKT, an endogenous ghrelin enhancer (80), exerts orexigenic effects by ghrelin secretion to stimulate food intake (71). It has been prescribed to treat nausea, vomiting, and anorexia (73). Moreover, RKT prevents plasma acylated-ghrelin levels from decreasing against cisplatin and increases appetite and food intake in the rat (81). RKT likely has an effect on activating ghrelin secretion and also reduces inactivation of ghrelin (82). A study with FD patients showed that a lower plasma des-acyl ghrelin level at baseline were associated with the higher efficacy of RKT (83). Also, RKT alleviated dyspeptic symptoms in FD patients with an increase of acylated-ghrelin levels (84). In addition, RKT was beneficial for aging-related decrease in food intake *via* ghrelin activity (85).

##### Effects on Gastric Motor Function

The effect of RKT on gastric relaxation is mediated by *β_2_*- and *β_3_*-adrenergic pathways, which are associated with smooth muscle relaxation (86, 87). Furthermore, RKT had a relaxant effect on fundus smooth muscles of isolated rat stomach, triggered by activation of the Ca^2+^-activated K^+^ channel (88). RKT has been shown to enhance gastric adaptive relaxation in the isolated guinea pig stomach (89).

RKT was shown to ameliorate delayed GE induced by N(G)-nitro-L-arginine (L-NNA), a nitric oxide (NO) synthase inhibitor, in rat. The hesperidin and L-arginine was identified as an active ingredient of RKT contributing to the increase in GE (90). Moreover, hesperidin, the major active component of RKT, was identified to stimulate contraction of intestinal smooth muscle mediated *via* 5-HT_3_ receptor pathway and acetylcholine release (75). The administration of RKT on normal and vagotomized dogs activated GI contractions during the interdigestive state and enhanced GE (91). In patients with FD, RKT improved gastric accommodation reflex and GE rate assessed by extracorporeal ultrasonography. In summary, RKT may be beneficial for treating GI dysmotility disorders, acting as a prokinetic agent (74).

##### Effects on Bile Salts

RKT showed a great binding capacity for bile salts and may prevent esophageal mucosal damage by bile acid exposure. It can be useful for the treatment of refractory GERD and duodeno-gastroesophageal reflux (92). This observation suggests that RKT may alleviate bile acid-induced mucosal hypersensitivity (93).

##### Effects on Stress-Induced Gastrointestinal Dysfunction

RKT can alleviate both GI and psychological symptoms in FD patients (94). First of all, physical or psychological stress can cause an imbalance in plasma ghrelin levels and decreasing gastric motility (95, 96). RKT may be beneficial for delayed GE induced by stress (97). Anxiety and stress can also induce dysfunction of the GI tract such as impairment of gastric accommodation (98, 99). RKT can modulate stress-induced gastric hypersensitivity and improving gastric accommodation (94). Moreover, it was shown that RKT improved GE through antagonistic activities on corticotropin-releasing factor receptor 1, 5-HT_2B/2C_ receptors, and 5-HT_3_ receptor in rats with delayed GE and anorexia model (75, 81, 100).

In a healthy human study to verify the effects of RKT on the hypothalamo-pituitary-adrenal (HPA) axis, RKT significantly suppressed plasma levels of adrenocorticotropic hormone (ACTH) and cortisol under continual stress, and improved the mental component of QoL (101). FD patients have an imbalance of autonomic nervous system function (102). RKT suppressed increased plasma levels of neuropeptide Y, a neurotransmitter of the sympathetic nervous system, under venipuncture stress (103). RKT also increased the activity of efferent vagus nerve and decreased the afferent activity of gastric vagus nerve, meanwhile, its active ingredient atractylodin stimulated ghrelin binding activity (104).

#### Clinical Studies

##### Functional Dyspepsia

In a randomized, placebo-controlled trial with 42 chronic idiopathic dyspepsia patients in Japan, RKT had a prokinetic action to improve GE, evaluated by the acetaminophen absorption method, and reduced upper GI symptoms (epigastric fullness, heartburn, belching, and nausea) compared to placebo (105) (**Table 3**). The first randomized, double-blind, placebo-controlled trial in Japan, 247 patients with FD by Rome III criteria, the administration of RKT for 8 weeks ameliorated symptoms of FD, especially epigastric pain and postprandial fullness. In addition, it showed a tendency whereby RKT was more effective among *H. pylori*-infected patients than the uninfected, providing a basis for different mechanisms of each symptom and treatment strategy depending on the of *H. pylori* infection (69). The Japanese DREAM study, a multi-center, randomized, double-blind, placebo-controlled trial, showed that RKT significantly improved dyspeptic but also psychological symptoms in 128 FD patients by Rome III criteria without *H. pylori* infection. After an 8 week RKT treatment, significant improvement was reported compared to placebo. RKT reduced upper GI symptoms especially postprandial fullness, early satiety, and bloating but also anxiety. Interestingly, the improvements of psychological symptoms were correlated with those of upper GI symptoms (106). In conclusion, RKT can be more useful for postprandial distress syndrome (PDS)-type of FD (110).

**Table 3:** Summary of clinical trials of rikkunshito.

Subject	Design	County	Comparison	Number	Outcome	Ref	Jadad score
Chronic idiopathic dyspepsia	Single-center, randomized, placebo-controlled	Japan	Rikkunshito 2.5 g t.i.d., a.c., 7 days *vs*. placebo (Combizym)	22:20	Rikkunshito increased gastric emptying. Rikkunshito reduced gastrointestinal symptoms.	Tatsuta et al. (105)	1
FD by Rome III	Multi-center, randomized, double-blind, placebo-controlled	Japan	Rikkunshito 2.5 g t.i.d., a.c., 8 weeks *vs*. placebo 2.5 g t.i.d., a.c., 8 weeks	125:122	Rikkunshito improved dyspeptic symptoms, such as epigastric pain and postprandial fullness.	Suzuki et al. (69)	5
FD by Rome III without *H. pylori* infection, severe heartburn, and depression	Multi-center, randomized, double-blind, placebo-controlled	Japan	Rikkunshito 2.5 g t.i.d., 8 weeks *vs*. placebo 2.5 g t.i.d., 8 weeks	64:61	Rikkunshito alleviated dyspeptic and psychological symptoms at the same time.	Tominaga et al. (106)	4
PPI-refractory GERD	Multi-center, randomized, parallel comparative	Japan	Rikkunshito 2.5 g t.i.d. + rabeprazole 10 mg q. d. 4 weeks *vs*. rabeprazole 20 mg qD 4 weeks	48:51	Improvement effect of rikkunshito combined with a standard dose of PPI was similar with double dose of PPI.	Tominaga et al. (107)	3
PPI-refractory NERD	Multi-center, randomized, double-blind, placebo-controlled	Japan	Rikkunshito 2.5 g t.i.d. + rabeprazole 10 mg q.d. 8 weeks *vs*. placebo 2.5 g t.i.d. + rabeprazole 10 mg q.d. 8 weeks	109:108	Rikkunshito combined with PPI improved mental health in non-obese patients and acid-related dysmotility symptoms in female and the elderly.	Tominaga et al. (73)	5
PPI-refractory GERD	Multi-center, open-labeled	Japan	Rikkunshito 2.5 g t.i.d. + PPI (rabeprazole 20 mg q.d. lansoprazole 30 mg q.d. omeprazole 20 mg q.d.) 6–8 weeks	47	Rikkunshito combined with PPI reduced dyspeptic symptoms and improved QoL on eating and sleep.	Kawai et al. (108)	0
PPI-refractory LPR	Single-center, randomized, parallel comparative	Japan	Rikkunshito 2.5 g t.i.d. 4 weeks *vs*. Rikkunshito 2.5 g t.i.d. + lansoprazole 30 mg q.d. 4 weeks	11:11	Rikkunshito reduced globus sensation. Rikkunshito enhanced delayed gastric emptying.	Tokashiki et al. (109)	2

FD, functional dyspepsia; GERD, gastroesophageal reflux disorder; PPI, proton pump inhibitor; NERD, non-erosive reflux disease ;q.d., once a day;t.i.d., three times a day; a.c., before meal; QoL, quality of life; LPR, laryngopharyngeal reflux.

##### Gastroesophageal Reflux Disease

A randomized, parallel comparative trial in Japan with 104 PPI-refractory GERD patients showed that the effect of RKT with standard dose of PPI on decreasing acid-related dysmotility symptom and reflux symptom was similar to that of a double dose of PPI. Particularly, this effect was greater in male NERD patients and in NERD patients with low body mass index (BMI < 22) (107). In a multi-center, randomized, double-blind, placebo-controlled study with 242 Japanese PPI-refractory NERD patients, the RKT combination group (standard dose of PPI plus RKT) improved the mental QoL component compared to the placebo group (standard dose of PPI plus placebo) at 4 weeks. However, the improvements of GERD symptoms were not significantly different between these groups. Through subgroup analysis, it was especially noteworthy that the mental-related scores in non-obese patients (BMI < 22) and acid-related dysmotility symptoms in female and the elderly (≥ 65 years) were more improved in the 8 week RKT group (73). It can also be suggested that RKT is more effective with postprandial symptoms (heavy feeling in stomach, sick feeling, and heartburn after meals) in elderly NERD patients (111). In addition, a clinical study conducted in 47 Japanese patients with PPI-refractory GERD treated for more than 8 weeks showed that the addition of RKT to PPI therapy for 6–8 weeks improved heartburn, fullness, abdominal discomfort, and abdominal pain as well as QoL for meals and sleep (108). Meanwhile, a randomized, parallel comparative trial conducted in 22 Japanese patients with PPI-refractory laryngopharyngeal reflux showed that the 4 week RKT treatment alleviated globus sensation, regardless of PPI co-administration. In addition, RKT enhanced delayed GE in positive correlation with the improvement of globus sensation (109).

#### Safety

In a meta-analysis of randomized controlled trials, there were few drug-related severe adverse events reported in the included studies (112). However, it is important to note that RKT should be administered by taking into account symptoms, age, pregnancy potential, and concomitant medications of the patient.

#### Usage

The standard RKT dose for adults in general practice is 7.5 g/day is containing 4.0 g of dried mixture consisting of eight crude herbs in fixed proportions. In Japan and several clinical trials, the standard RKT dose for adults in general practice is 7.5 g/day (2.5 g three times a day) with a proper volume of water before or between meals for 8 weeks (73, 74, 107). The dosage may be adjusted according to the patient’s age, body weight, and symptoms (106).

### DA-9701 (Motilitone^®^)

DA-9701 is a new herbal drug developed in South Korea that received New Drug Application approval in May 2011 from the Korean Food and Drug Administration (113). It is formulated with ethanolic extracts of Pharbitidis semen from the seeds of *Pharbitis nil* Choisy and Corydalis tuber from the roots of *Corydalis yanhusuo* W. T. Wang (114) These two herbs have been commonly used in traditional medicine in China, Korea, and Japan for abdominal and gynecological symptoms. Pharbitidis semen has been used as an analgesic for the abdomen and a stimulant of intestinal peristalsis (114). Corydalis tuber has been used as an analgesic or anti-spasmodic agent for the GI tract (115). Moreover, it has been known to have an effect on gastric secretion and prevention of ulcer (116). The active ingredients of DA-9701 include chlorogenic acid in Pharbitidis semen and corydaline and tetrahydropalmatine in Corydalis tuber. The pharmacological study has demonstrated that DA-9701 has dopamine D_2_ antagonistic activity, adrenergic α_2_ agonist activity, 5-HT_1A_ agonist activity, and 5-HT_4_ agonist activity (113, 114).

#### Mechanisms of Action

##### Effects on Gastrointestinal Motility

The oral administration of DA-9701 significantly increased semi-solid or solid GE in normal rat and mice (117, 118). Moreover, DA-9701 restored semi-solid or liquid GE in the apomorphine or cisplatin-induced delayed GE rat model (117). Immobilization induced delayed GE was also reversed by oral administration of DA-9701. A study with strain gauge force transducer in the antrum of rat showed that oral administration of DA-9701 improved the clonidine-induced hypomotility of the gastric antrum, but it showed no effect on antral motility in normal conditions (119).

The effect of GE in healthy volunteers was investigated using gastric magnetic resonance imaging in a randomized, double-blind, placebo-controlled trial (120). After administration of 60 mg of DA-9701 or placebo t.i.d. for 7 days, GE was significantly enhanced in DA-9701 group compared to the placebo group. In another randomized, double-blind, controlled trial with patients with Parkinson’s disease, 30 mg of DA-9701 three times per day before meals for 4 weeks significantly increased GE, while domperidone showed no effect (121). The concentration of plasma levodopa was increased in the DA-9701 group only, though it was not statistically significant.

DA-9701 also increased small intestinal motility in an animal study. *In vitro* study with ileal muscle strip of guinea pig showed that DA-9701 increased contractility in normal condition as well as morphine pre-treated hypomotility state (122). In postoperative ileus or atropine-induced delayed GI transit model of rat and guinea pig, oral administration of DA-9701 restores delayed transit (117, 122, 123). In contrast to the pathologic condition, only high dose of DA-9701 increased GI motility in the normal condition (117, 123). The DA-9701 seemed to influence active ghrelin levels in the stress or post-operative ileus rat model (123, 124). In addition, the central corticotropin-releasing factor pathway may mediate the improvement in GI transit and the inhibition of plasma ACTH levels by DA-9701 in the postoperative ileus guinea pig model (125).

##### Effects on Gastric Accommodation

An *in vivo* study with Beagle dogs showed that oral administration of DA-9701 induced proximal gastric relaxation and shift the pressure-volume curve to left similar to the intravenous administration of cisapride (117). Another study with Beagle dog showed that oral administration of DA-9701 induced gastric accommodation dose-dependently during the postprandial phase similar to the oral administration of sumatriptan (126). Restraint stress-induced feeding inhibition was reversed by oral DA-9701, and this effect was blocked by a 5-HT_1A_ antagonist in a rat study (127). A tissue bath study using rat gastric muscle strip suggested that the nitrergic rather than the purinergic pathway was involved in gastric accommodation by DA-9701 (128).

##### Effects on Colonic Contractility

In a tissue bath study, DA-9701 did not influence the contractility of normal colonic muscle strips; however, it increased the motility of distal colonic muscle strips of a morphine pre-treated hypomotility state (122). DA-9701 increased fecal pellet output in the *in vivo* morphine-induced constipation guinea pig model and improved defecatory dysfunction in acute spinal shock state in spinal cord injury rat model (122, 129).

##### Effect on Visceral Sensitivity

DA-9701 did not affect visceral perception in the normal rat. However, in the rat with visceral hypersensitivity, which was induced by neonatal colonic irritation, DA-9701 significantly decreased pain threshold in a dose-dependent manner (130).

#### Clinical Studies

##### Functional Dyspepsia

Two large multicenter clinical trials for FD of DA-9701 have been conducted in South Korea using different Rome criteria for FD and efficacy assessment methods (131, 132) (**Table 4**). A randomized, controlled, multi-center study with 462 Korean FD patients by Rome II criteria showed that the effect of 30 mg DA-9701 three times a day on the FD symptom was similar to 50 mg itopride hydrochloride three times a day (131). The overall responder rates were 37% for DA-9701 and 36% for the itopride group at 4 weeks. DA-9701 and itopride significantly reduced the score of all individual symptoms (upper abdominal pain, upper abdominal discomfort, epigastric burning, inability to finish a regular meal, fullness after eating, pressure in the upper abdomen, bloating, and nausea) from baseline. Another randomized, double-blind, multi-center study with 389 Korean FD patients by Rome III criteria showed that the effect of 30 mg DA-9701 three times a day on the FD symptom was similar to 40 mg pantoprazole once-daily (132). The global symptom improvement rates were 60.5 and 65.6% in the DA-9701 and pantoprazole groups at 4 weeks, respectively. Both DA-9701 and pantoprazole significantly reduced the score of all individual symptoms (epigastric pain epigastric soreness score, early satiety score, and postprandial fullness) from baseline. Interestingly, combination therapy of DA-9701 and pantoprazole did not increase the response rate compared with DA-9701 or pantoprazole monotherapy. Both studies demonstrated that DA-9701 therapy significantly improved symptom-related QoL in patients with FD.

**Table 4:** Summary of clinical trials of DA-9701.

Subject	Design	County	Comparison	Number	Outcome	Ref.	Jadad score
FD by modified Rome II	Multi-center, randomized, double-blind, controlled	Korea	DA-9701 30 mg t.i.d., a.c., 4 weeks *vs*. Itopride 50 mg t.i.d., a.c., 4 weeks	228:227	DA-9701 and itopride significantly improved symptoms and QoL. DA-9701 was not inferior to itopride for symptom improvement.	Choi et al. (131)	5
FD by Rome III	Multi-center, randomized, double-blind, controlled	Korea	Placebo PPI q.d. a.c. + Motilitone 30 mg t.i.d., a.c., 4 weeks *vs*. Pantoprazole 40 mg q.d. a.c. + Placebo t.i.d., a.c., 4 weeks *vs*. Pantoprazole 40 mg q.d. a.c.+ Motilitone 30 mg t.i.d. a.c. 4 weeks	131:131:127	Symptoms and QoL were significantly improved with no significant difference among three groups.	Jung et al. (132)	5
FD by Rome III with *H. pylori*-positive	Multi-center, randomized, double-blind, placebo-controlled	Korea	DA-9701 30 mg t.i.d., a.c., 12 weeks *vs*. Eradication therapy 1 week + placebo 11 weeks	12:18	Effect of DA-9701 therapy on FD symptom was not different from the eradication group.	Park et al. (133)	5
Minimal change with reflux and dyspeptic symptoms	Bi-center, randomized, double-blind, placebo-controlled	Korea	DA-9701 30 mg t.i.d. 4 weeks *vs*. placebo	42:39	Symptoms and QoL were improved; however, DA-9701 was not superior to placebo.	Park et al. (134)	4
Parkinson’s disease	Single center, randomized, double-blind, controlled	Korea	DA-9701 30 mg t.i.d., a.c., 4 weeks *vs*. Domperidone 10 mg t.i.d., a.c., 4 weeks	19:19	DA-9701 significantly increased GE, but not domperidone. The concentration of plasma levodopa was increased in DA-9701 group only, but not significant.	Shin et al. (121)	5
FC by Rome III	Single center, open-labeled	Korea	DA-9701 30 mg t.i.d., a.c., 24 days	33	Constipation-related symptoms were all significantly improved after treatment. Right and rectosigmoid colon transit time was significantly decreased.	Kim et al. (135)	1

FD, functional dyspepsia; FC, functional constipation; q.d., once a day; t.i.d., three times a day; a.c., before meal; QoL, quality of life; GE, gastric emptying.

*H. pylori* infection is considered a possible cause of FD symptoms. In sub-analysis for the *H. pylori*-positive group in the above clinical trial comparing DA-9701 and pantoprazole, the response rate was significantly higher in the pantoprazole alone and combination therapy group compared with the DA-9701 alone group (132). While in a small study with 30 patients with FD by Rome III criteria and *H. pylori*-positive, DA-9701 therapy group showed higher symptom improvement rates at 12 weeks compared with eradication group (73.3 and 60%, respectively); however, it was not statistically different because of the small sample number (133).

##### Gastroesophageal Reflux Disorder

A randomized, double-blind, placebo-controlled study for the effect of DA-9701 on GERD was conducted in 81 patients with minimal change esophagitis presenting with reflux and dyspeptic symptoms (134). Although the Nepean dyspepsia index and QoL were improved after 4 weeks, 30 mg DA-9701 three times a day was not superior to placebo. The outcome might have been associated with the high placebo response in patients with NERD or FD (134). In subgroup analysis, DA-9701 significantly improved the reflux symptom score compared to the placebo in patients aged 65 years or older (134).

##### Constipation

DA-9701 has an affinity for the 5-HT_4_ receptor, and some animal studies have showed it increases colonic motility (122, 129). A prospective and single-center study investigated the efficacy of DA-9701 in 27 patients with functional constipation by Rome III criteria (135). After administration of 30 mg DA-9701 three times a day for 24 days, spontaneous bowel movement, stool form, and constipation-related subjective symptoms were improved. Moreover, right and rectosigmoid colon transit time significantly decreased. However, this study was an open-labeled, uncontrolled study, and most participants were young-aged women (mean age 36.1 ± 15.4 years, 93% female).

#### Safety

The incidence of adverse events of DA-9701 was not different from itopride or pantoprazole in clinical trials. The reported adverse events include nausea, diarrhea, vomiting, constipation, pruritus, and increased alanine aminotransferase or prolactin levels with mild severity (131, 132). There was no clinically significant cardiovascular events in clinical studies.

#### Usage

In two large clinical trials, the standard dose of DA-9701 (Motilitone^®^, Dong-A ST, Seoul, Korea) for adults was 30 mg three times a day before meals.

## Summary and Conclusions

STW-5 normalizes the stomach and intestinal motility and reduces inflammation and gastric acid production. These effects partially influence the pathophysiology of the FGIDs and their therapeutic effects have been proven in several clinical studies. However, despite the animal experiments that showed fundal relaxation and improvement in antral hypomotility by STW-5, at least ≥50% of patients with FD did not have delayed gastric emptying, and more patients had no abnormalities of gastric accommodation. Although STW-5 reduces the secretion of gastric acid, gastric acid hypersecretion is not always observed in patients with FD. Therefore, such therapeutic effects on the motility and other functions of the GI tract may not necessarily lead to improvement of FGID symptoms, and well-designed clinical studies are still lacking. However, as STW-5 has multiple mechanisms of action and show favorable safety profiles, further studies on their roles on pathophysiology of FGIDs will allow the use of STW-5 as a promising alternative treatment for FGIDs, especially FD and IBS.

Evidence from *in vitro* studies and clinical trials indicate that PMO seems to alleviate IBS symptoms, mainly abdominal pain, by relaxing smooth muscles in the gut. A recent meta-analysis examining 12 randomized controlled trials showed that PMO was beneficial in the management of IBS and an Asian consensus on IBS mentioned its potential efficacy in treating IBS with a high level of agreement (8, 63). However, any benefit of PMO remains unclear in other FGIDs such as FD owing to the paucity of reliable preclinical and clinical data. Furthermore, relatively unclear mechanisms of action of the active ingredients, unstandardized formulation, and unidentified adverse events are currently significant challenges for its use in the treatment of FGIDs. Relevant evidence based on rigorous studies supporting the efficacy and safety of PMO is needed.

According to a meta-analysis of randomized controlled trials (112), the effects of RKT on the treatment of FD were better than prokinetic drugs, though there was lack of clinically significant evidence due to poor quality of the included studies such as selection bias. Nevertheless, basic research and clinical studies have elucidated that RKT improves esophageal clearance and motility, and promotes gastric motor activity including gastric accommodation and emptying. It also increases ghrelin secretion and food intake. Moreover, RKT attenuates stress-induced injury on the GI tract *via* the brain-gut axis and balancing the autonomic nervous system. Consequently, it is possible and clinically meaningful to target RKT for subtypes of FD (e.g., PDS type) or specific symptoms (e.g., postprandial fullness), and co-administration with PPI to resolve symptoms of refractory GERD that do not respond to conventional PPI treatment may be more effective.

Several preclinical and human studies have demonstrated that DA-9701 increased gastric emptying and antral motility in normal or diseased states. In addition, DA-9701 improved gastric accommodation as well as feeding inhibition by stress. Based on these results, subsequent clinical trials in patients with FD demonstrated that DA-9701 was not inferior compared with itopride or PPI to improve FD symptoms. It was also suggested that DA-9701 could improve symptoms of GERD and constipation in the specific patient population. However, most studies for the effect of DA-9701 on FD and other FGIDs have performed only in one country. Moreover, the clinical trials for FD recruited a large number of participants but were non-inferiority studies. Therefore, well-designed clinical trials in different countries and more detailed mechanical studies should be performed in the future.

The role of herbal therapies in FGIDs is still unclear. The active ingredients and mechanisms of action have not been fully identified and well-designed clinical trials are insufficient. However, herbal therapies play a role as a complementary and alternative medicine and may find suitable application in refractory patients. The development of various promising herbal medications in the near future may help to improve the QoL of patients with FGIDs.

## Author Contributions

YK, J-WK, JK and HR conceptualized the study. YK, J-WK, N-YH and HR wrote the first draft of the manuscript. YK, J-WK and JK critically revised the manuscript. HR received the grant. All authors contributed to the article and approved the submitted version.

## Funding

This paper was supported by Wonkwang University in 2019.

## Conflict of Interest

The authors declare that the research was conducted in the absence of any commercial or financial relationships that could be construed as a potential conflict of interest.
